# Experiments on the Operation of Morphine in the Human Body

**Published:** 1829-07-01

**Authors:** 


### XLVIII.

### Experiments on the Operation of Morphine in the Human Body in a State of Health. By Dr. Beraudi, of Turin.

It is now some twenty years since a talented physician still practising in the modern Athens, was quizzed a little in consequence of entertaining a number of his brethren, once or twice a week, —not with tea and cards,—but with tincture of digitalis! The parties assembled, and commenced the beverage, each keeping a finger on the pulse of his neighbour, in order to determine the physiological operation of foxglove. The result is well known. Digitalis was proved by *direct experiment*, to be a strong stimulant—although since that period the profession has doggedly adhered to the vulgar opinion that the drug is a sedative, and employ it accordingly. A somewhat similar party was lately formed at Turin, consisting of Messrs. Beraudi, Rebrini, Crispo, and Allinio, for the purpose of taking the acetate of morphium, and thus ascertaining its effects on people in health. These four gentlemen met on the 28th of September last, having previously dined, and commenced their experiments. At three o'clock M. Allinio, aged 22 years, of bilious temperament, and whose pulse was at 66, swallowed an eighth of a grain of the acetate in some distilled water. He had scarcely taken the medicine when he felt a bitter and somewhat acrid taste in the back of the throat. In five minutes, there was severe pain in the epigastrium—propensity to sleep, with somewhat laborious breathing. At the end of twenty-five minutes, the same phenomena continued. At 30 minutes, there was profuse perspiration—with dilated pupils—and pulse at 94. At 33 minutes, there was heavy drowsiness, with pain about the frontal sinuses. At 50 minutes, the lips were livid, the face flushed, the conjunctivæ injected, severe pain in the frontal bone. At 52 minutes, pains in the bladder, physiognomy stupid, eyes sparkling, urgent thirst, sense of extreme lassitude in the inferior extremities. At a quarter past 4 o'clock, there was pruritus of the skin, continual pains in the genito-urinary organs—weight in the forehead. These symptoms continued till nearly seven o'clock, when severe pain in the epigastrium was followed by vomiting. No sleep took place till two o'clock the next morning, when the experimenter fell into profound repose till six, when he awoke with obtuse pain in the head, and soon afterwards had two alvine evacuations.

The other three gentlemen took the acetate, some in larger and some in smaller doses, at the same hour. Two of them were affected in a manner not particularly different from that already described. One of them, however, experienced very little else than an acceleration of the pulse to 108 in the minute. In the course of a couple of days the experiments were repeated, but on an empty stomach. The symptoms were not precisely those which followed the medicine taken after food, but yet they were not materially different, and need not be detailed. The experiments were afterwards repeated with still larger doses of the medicine, and a corresponding degree of intensity in the symptoms. We are not aware that much useful information is to be collected from these experiments. The Northern digitalis-sippers came to the conclusion that foxglove was a stimulant—if we may judge by the symptoms above described, the Italian morphium-eaters have a fair right to infer that morphine is an irritant—for certainly its effects were any thing but soothing.

One thing we would hint to our juvenile experimenters, namely, that medicines when taken *in health* produce very different effects from those which result from the same remedies taken *in diseases*.

